# Doxycycline reduces liver and kidney injuries in a rat hemorrhagic shock model

**DOI:** 10.1186/s40635-023-00586-4

**Published:** 2024-01-09

**Authors:** Regina Sordi, Luana Bojko, Filipe R. M. B. Oliveira, Thiele Osvaldt Rosales, Camila Fernandes Souza, Lucas Wenceslau Moreno, Gustavo Ferreira Alves, José Carlos Rebuglio Vellosa, Daniel Fernandes, Jose Rosa Gomes

**Affiliations:** 1 Department of Pharmacology, Graduate Program in Pharmacology, Universidade Federal de Santa Catarina, Florianópolis, SC Brazil; 2 Department of Structural Biology, Molecular and Genetics, Universidade Estadual de Ponta Grossa, Ponta Grossa, PR Brazil; 3 Department of Clinical and Toxicological Analysis, Universidade Estadual de Ponta Grossa, Ponta Grossa, PR Brazil; 4 Department of Structural Biology, Molecular and Genetics, Post Graduation Program in Biomedical Science, Universidade Estadual de Ponta Grossa, Avenida Carlos Cavalcanti, 4748, Ponta Grossa, PR 84030-900 Brazil

**Keywords:** Hypovolemic shock, Hemorrhage, Nitric oxide, Creatinine, Drug repurposing, Organ failure

## Abstract

**Background:**

Hemorrhagic shock (HS), which causes insufficient tissue perfusion, can result in multiple organ failure (MOF) and death. This study aimed to evaluate whether doxycycline (DOX) protects cardiovascular, kidney, and liver tissue from damage in a rat model of HS. Immediately before the resuscitation, DOX (10 mg/kg; i.v.) was administered, and its protective effects were assessed 24 h later. Mean arterial pressure, renal blood flow, heart rate, vasoactive drug response, and blood markers such as urea, creatinine, AST, ALT, CPK, CPR, and NOx levels were determined.

**Results:**

We showed that DOX has a significant effect on **r**enal blood flow and on urea, creatinine, AST, ALT, CPK, and NOx. Morphologically, DOX reduced the inflammatory process in the liver tissue.

**Conclusions:**

We conclude that DOX protects the liver and kidney against injury and dysfunction in a HS model and could be a strategy to reduce organ damage associated with ischemia-and-reperfusion injury.

## Background

Multiple organ failure can be the result of trauma injuries, which is one of the main causes of mortality among young people and adults, resulting in roughly 5 million deaths each year [1], with half of these deaths caused by severe hemorrhage and hemorrhagic shock (HS). During HS, the reduction of blood volume causes insufficient tissue perfusion, resulting in an imbalance between oxygen consumption and supply, which can lead to multiple organ failure (MOF) [2].

Blood levels of several molecules that are used to identify injuries and dysfunctions, such as urea, creatinine, aspartate aminotransferase (AST), alanine aminotransferase (ALT), creatine phosphokinase (CPK), C-reactive protein (CRP), and nitrite and nitrate (NOx), may be monitored to verify if any organ injuries have taken place [3]. Additionally, other molecules, such as matrix metalloproteinases, are often produced by inflammatory cells and have an important role in the process of organ injury [4].

Unfortunately, there is no specific treatment to prevent or minimize MOF associated with HS [5]. As a result, researchers are interested in investigating the effects of approved drugs, at least in experimental models of HS [6–9].

In this context, doxycycline (DOX), a member of the tetracycline family of antibiotics used to treat various bacterial infections, has been exhibiting potential clinical benefits. It also exhibits pleiotropic effects, including anti-inflammatory and antioxidant properties [10, 11]; suppresses nitric oxide (NO) overproduction [12, 13], and matrix metalloproteinases (MMPs) activity. Both molecules (NO and MMPs) are strongly related to poor outcomes in severe situations including sepsis and HS [3, 14, 15].

It's interesting to note that DOX effects have been studied in both human and animal models for a variety of diseases like cardiac disease, arthritis, periodontitis, and chronic wounds [11, 14, 16, 17] that are characterized by increased MMP activity and inflammation.

Despite DOX has been tested in animal models of sepsis and even HS-related MOF [3, 18], its effects in MOF associated with a recovery model of HS, particularly in the morphology of important organs such as the liver and kidney are unknown.

Therefore, using our stablished protocol to create an experimental HS condition [7, 8, 19, 20], the aim of this study was to evaluate the protective effect of DOX when administered at the resuscitation in a rat model of HS.

## Methods

### Animals

Male Wistar rats (200–250 g) were housed in a temperature- and light-controlled room (23 ± 2 °C; 12 h light/dark cycle) with free access to water and food. Five rats per cage were kept in 45 × 34 × 16 cm plastic cages. All experiments were performed between 9:00 and 16:00 h. Animal procedures are in accordance with the National Institutes of Health Animal Care Guidelines and the Guide of the Brazilian National Council of Animal Experimentation. Animal studies are reported in compliance with the ARRIVE guidelines. The procedures were approved by the Universidade Estadual de Ponta Grossa Ethics Committee (protocol number 048/2015).

### Hemorrhagic shock procedure and experimental groups

The HS model was performed as previously described with minor modifications [7, 8]. Rats were anesthetized with sodium thiopentone (100 mg/kg i.p.). Left femoral artery (for blood withdrawn and blood pressure measurement) and vein (for resuscitation and drug delivery) were cannulated (polythene tubing; PP25; Smiths Medical International Ltd, Kent, UK). Blood was withdrawn from the artery (at a rate of 1 mL/min) and collected with heparin (2 IU per mL of blood; Hepamax- Blausiegel, Cotia, SP, Brazil) until the mean arterial pressure (MAP) reached 40 ± 2 mmHg, which was maintained for 90 min either by further withdrawal of blood during the compensation phase or administration of the shed blood during the decompensation phase. Blood pressure was recorded with a catheter pressure transducer coupled to a Powerlab 4/30 (ADInstruments Pty Ltd.; Castle Hill, New South Wales, Australia) running the proprietary software LabChart 8^®^. The shed blood was kept between 6 and 10 °C. Immediately before the resuscitation with blood, HS animals were randomly divided to receive vehicle (saline; 1 mL/kg; i.v.) or doxycycline (DOX; 10 mg/kg/mL; i.v.) *in bolus* through the femoral vein. Then, resuscitation was performed with the shed blood (warmed to 22 ± 2 °C) over a period of 5 min followed by 1.5 mL/kg of Ringer’s lactate (Eurofarma-Ribeirão Preto, SP, Brazil). Twenty minutes after the end of resuscitation, cannulas were removed, vessels were ligated, and the skin was sutured. All animals were allowed to recover from anaesthesia in a warm cage. Sham-rats were used as control and underwent identical surgical procedures, but without haemorrhage or resuscitation, and were also randomly treated with DOX or saline. Twenty-four hours after resuscitation, recovery animals were anesthetized once more for assessment of all parameters described below. One group of animals underwent cardiovascular analyses (mean arterial pressure, heart rate, and response to vasoactive drugs). A second group of animals underwent renal blood flow determination, as well as the extraction of liver and blood samples as detailed below.

### Cardiovascular parameters

In the first set of experiments, cardiovascular parameters were determined in sham and HS rats. Twenty-four hours after HS or sham surgery, animals were anesthetized with sodium thiopentone at dose 100 mg/kg i.p. (Cristalia—Itapira, SP, Brazil), and the jugular vein (for drug administration) and right carotid artery (for blood pressure measurement) were cannulated. MAP and heart rate (HR) data were recorded with a catheter pressure transducer coupled to a Powerlab 4/30 (ADInstruments Pty Ltd.; Castle Hill, New South Wales, Australia). Baseline MAP and HR were determined through the software LabChart 8^®^. In addition, rats were injected intravenously with acetylcholine (ACh) 0.1, 1, 10 nmol/kg, sodium nitroprusside (SNP) 3, 10, 30 nmol/kg), phenylephrine (Phe) 3, 10, 30 nmol/kg) and angiotensin II (Ang II) 3, 10, 30 pmol/kg (Sigma-Aldrich Company Ltd, Poole, Dorset, UK). The change in MAP (area under the curve from baseline—arbitrary units; AU) was calculated through the LabChart 8^®^ software. Although a dose–response curve has been performed, for the sake of clarity, only the intermediate dose of each drug is shown in the graphs. At the end of the experiments, animals received an overdose of anaesthetic.

### Renal blood flow (RBF)

In a separated group of animals, animals were anesthetized with sodium thiopentone at dose 100 mg/kg i.p. (Cristalia—Itapira, SP, Brazil). An abdominal incision was done to assess the left kidney, and a laser probe (model VP3) connected to a laser Doppler blood flow monitor (moorVMSLDF2, Moor Instruments, England) was placed directly on the kidney surface. Baseline RBF was determined through the software LabChart 8^®^. Immediately after RBF measurement, blood from all animals was obtained through cardiac puncture (exsanguination), and the livers were harvested. Blood was centrifuged (3000 g, 10 min) and the plasma was obtained and kept at −80 °C for the analyses described below.

### Markers of organ injury and dysfunction

Plasma was obtained for urea, creatinine, aspartate aminotransferase (AST), alanine aminotransferase (ALT) and creatine phosphokinase (CPK) measurement through commercially available clinical assay kits (LabTest Diagnóstica S.A., Lagoa Santa, MG, Brazil). Plasma C-reactive protein (CRP) was quantified through a highly sensitive rat enzyme-linked immunosorbent assay kit (Immunology Consultants Laboratory Inc.; Newberg, OR, USA). The results were expressed as mg/mL. Plasma nitrite and nitrate (NOx) were zinc sulfate-deproteinized and subjected to nitrate conversion to nitrite using *Escherichia coli* nitrate reductase for 3 h at 37 °C. Samples were centrifuged for bacteria removal, and 100 µL of each sample was mixed with Griess reagent (1% sulphanilamide in 10% phosphoric acid / 0.1% naphtylethylenediamine) in a 96-well plate and read at 540 nm in a plate reader. Standard curves of nitrite and nitrate were run simultaneously, and values were expressed as µM.

### Histology

Twenty-four hours after surgery, liver tissues were harvested, fixed (paraformaldehyde 4%) and embedded in paraffin. Sections (5 µm) were cut and stained with hematoxylin–eosin (HE) and examined under light microscopy. To quantify inflammation in liver tissue, 10 random fields (1000 ×) were evaluated, and polymorphonuclear (PMN) cells were counted.

### Drugs

Unless otherwise stated, all compounds were from Sigma-Aldrich Company Ltd (Poole, Dorset, UK). Ringer’s lactate was from Eurofarma-Ribeirão Preto, SP, Brazil); sodium thiopentone (Thiopentax) from Cristalia (Itapira, SP, Brazil); heparin (Hepamax) from Blausiegel (Cotia, SP, Brazil).

### Statistical analysis

The GPower 3.1.1 software was used for sample calculation, which was based on the SD and the magnitude of difference between the groups obtained in the analysis of MAP (mmHg) from our previous studies [8]. Considering 4 experimental groups, alpha = 0.05, and a power of 80%, 7 animals were required for statistical significance. As a certain mortality rate was expected, 10 animals were assigned per group, and the final number (n) in each group is indicated in the figures. When necessary, the values were transformed into logarithms to achieve normality and homogeneity of variances, verified by the Shapiro–Wilk and Bartlett tests, respectively. The results are presented as dot plot showing the mean ± SD. Statistical significance (p < 0.05) was analysed by ANOVA followed by Tukey’s post hoc test as indicated in the legends of figures. Pearson correlations were computed to examine the strength of associations in normally distributed samples (Fig. 2B–E). The statistical analyses were performed using GraphPad Prism^®^ version 9.0 software (California, USA).

## Results

### Doxycycline attenuated renal dysfunction and liver injury

**Fig. 1:**
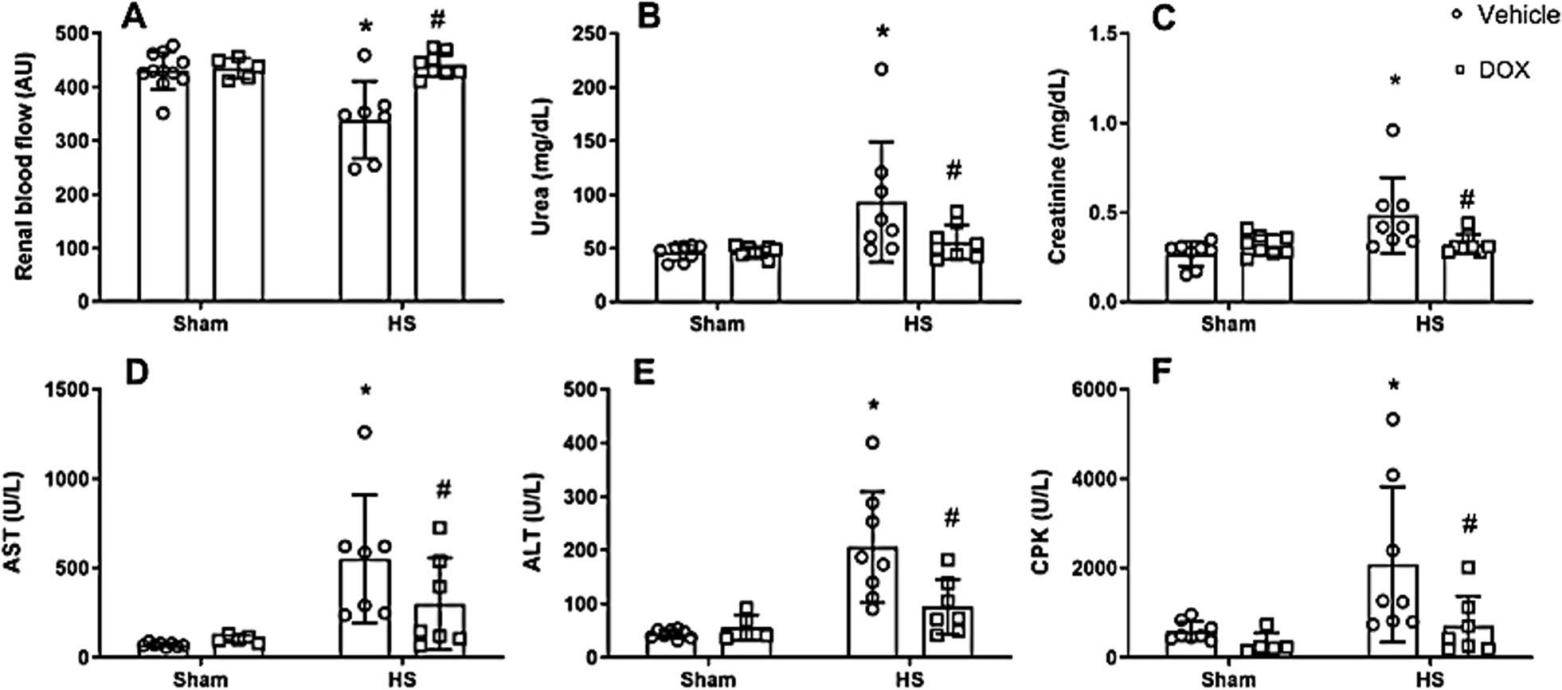
Measurement for renal blood flow (**A**); plasma urea (**B**); plasma creatinine (**C**); plasma aspartate aminotransferase (AST) (D); plasma alanine aminotransferase (ALT) (**E**); and CPK, plasma creatine phosphokinase (**F**) in control groups (Sham) and HS treated with vehicle or doxycycline. *P < 0.05 vs. sham + vehicle, and ^#^P < 0.05 vs. HS + vehicle

When compared with sham rats, HS animals exhibited a decrease in RBF (Fig. 1A), along with elevated levels of plasma urea (Fig. 1B) and creatinine (Fig. 1C), suggesting renal dysfunction. HS rats also developed liver injury characterized by increased levels of AST (Fig. 1D) and ALT (Fig. 1E), and muscular injury, as observed by increase of CPK (Fig. 1F). The treatment of HS rats with DOX prevented the decrease of RBF and reduced the plasma markers of organ damage.

### Doxycycline reduced significantly NOx production in HS rats

**Fig. 2:**
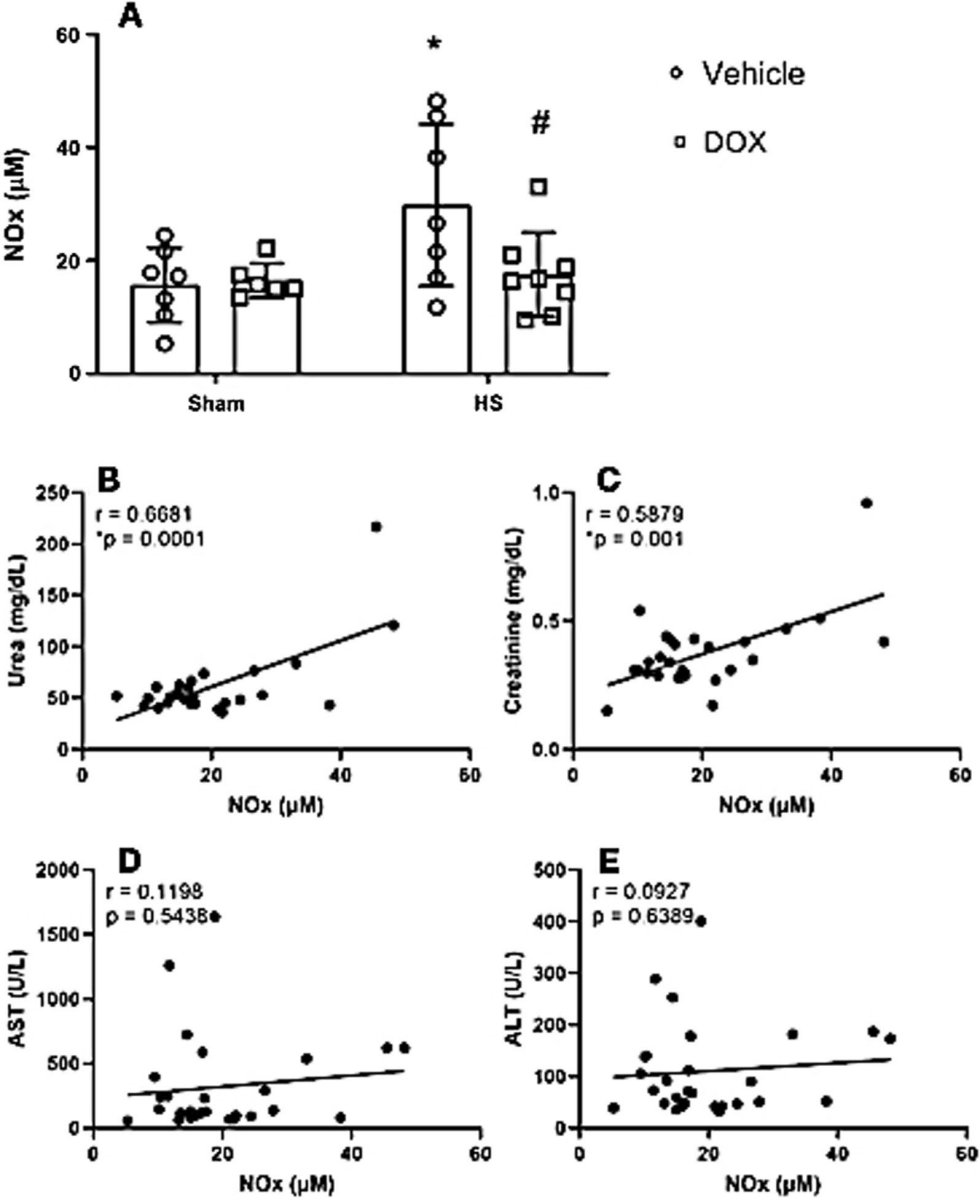
Measurements for NOx production (**A**) and its correlations with urea (**B**), creatinine (**C**), plasma aspartate aminotransferase (AST) (**D**); plasma alanine aminotransferase (ALT) in control groups (Sham) and HS treated with doxycycline. *P < 0.05 vs. sham + vehicle, and ^#^P < 0.05 vs. HS + vehicle

Rats in the HS group had significantly increased NOx levels compared to rats in the Sham control group (Fig. 2A). The administration of DOX in HS rats decreased NOx levels when compared to HS vehicle group. Additionally, the NOx levels showed a positive correlation (p < 0.05) with urea and creatinine (Fig. 2B, C), but not with AST, ALT (Fig. 2D, E) or CPK (data not shown).

### Doxycycline had no effect on mean arterial pressure (MAP), heart rate (HR) in HS rats, or C-reactive protein (CRP) levels

**Fig. 3:**
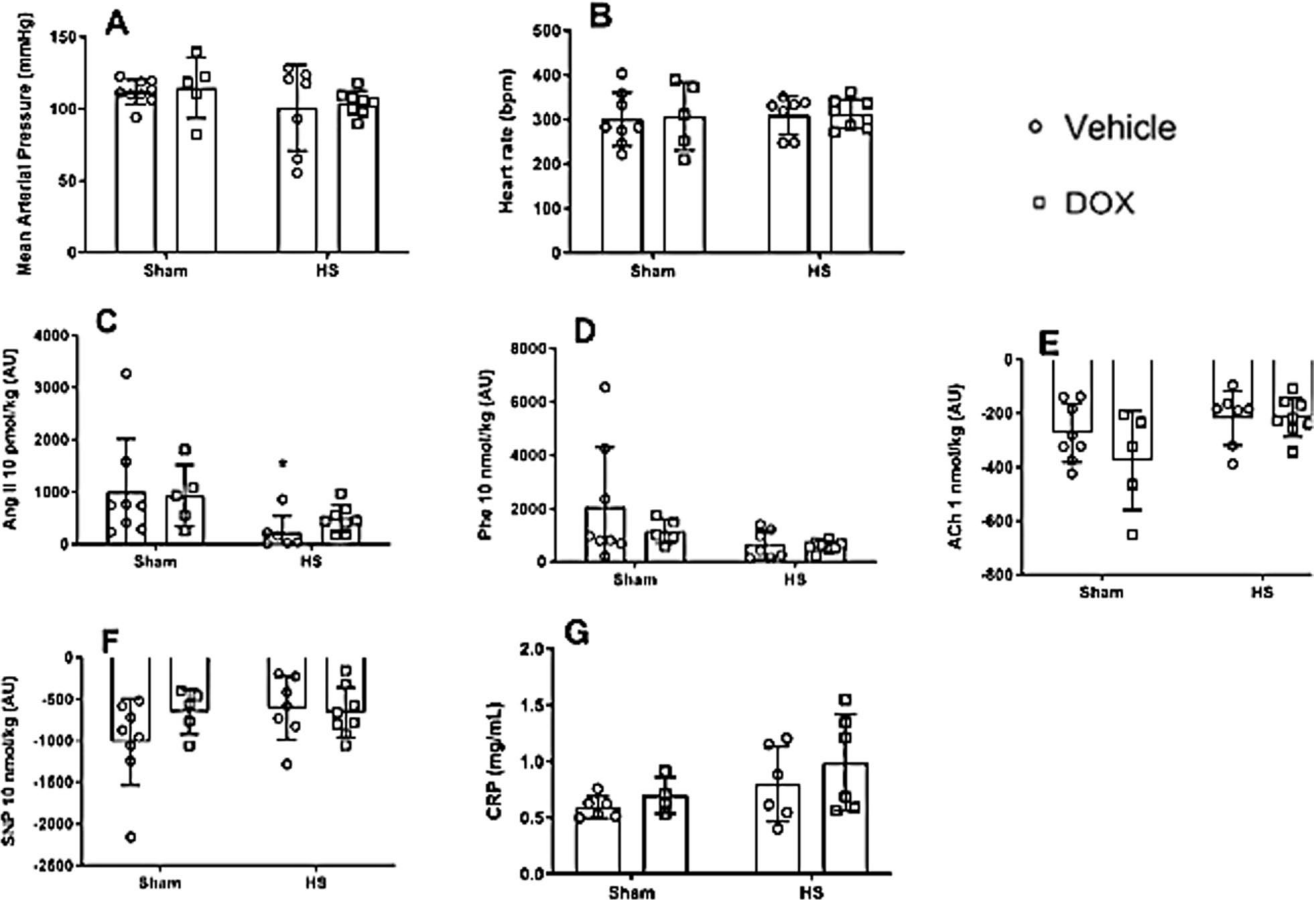
Measurement for mean arterial pressure (mmHg) (**A**), heart rate (bpm) (**B**), response to angiotensin II (Ang II; 10 pmol/kg) (**C**), response to phenylephrine (Phe; 10 nmol/kg) (**D**), response to acetylcholine (ACh; 1 nmol/kg) (**E**), response to sodium nitroprusside (SNP; 10 nmol/kg) (**F**) and for C-reactive protein (**G**) in control groups (Sham) and HS treated with doxycycline. *P < 0.05 vs. sham + vehicle, and ^#^P < 0.05 vs. HS + vehicle

Twenty-four hours after HS, the baseline values of MAP (Fig. 3A) and HR (Fig. 3B) were similar in all experimental groups. However, the HS group showed a significant hypo-responsiveness to Ang II (Fig. 3C), and DOX therapy for HS rats failed to restore this response. Additionally, there was no difference among the groups for Phe, ACh, SNP responses, and for CRP levels (Fig. 3D–G, respectively).

### Doxycycline reduced the inflammation process in liver of HS rats

**Fig. 4:**
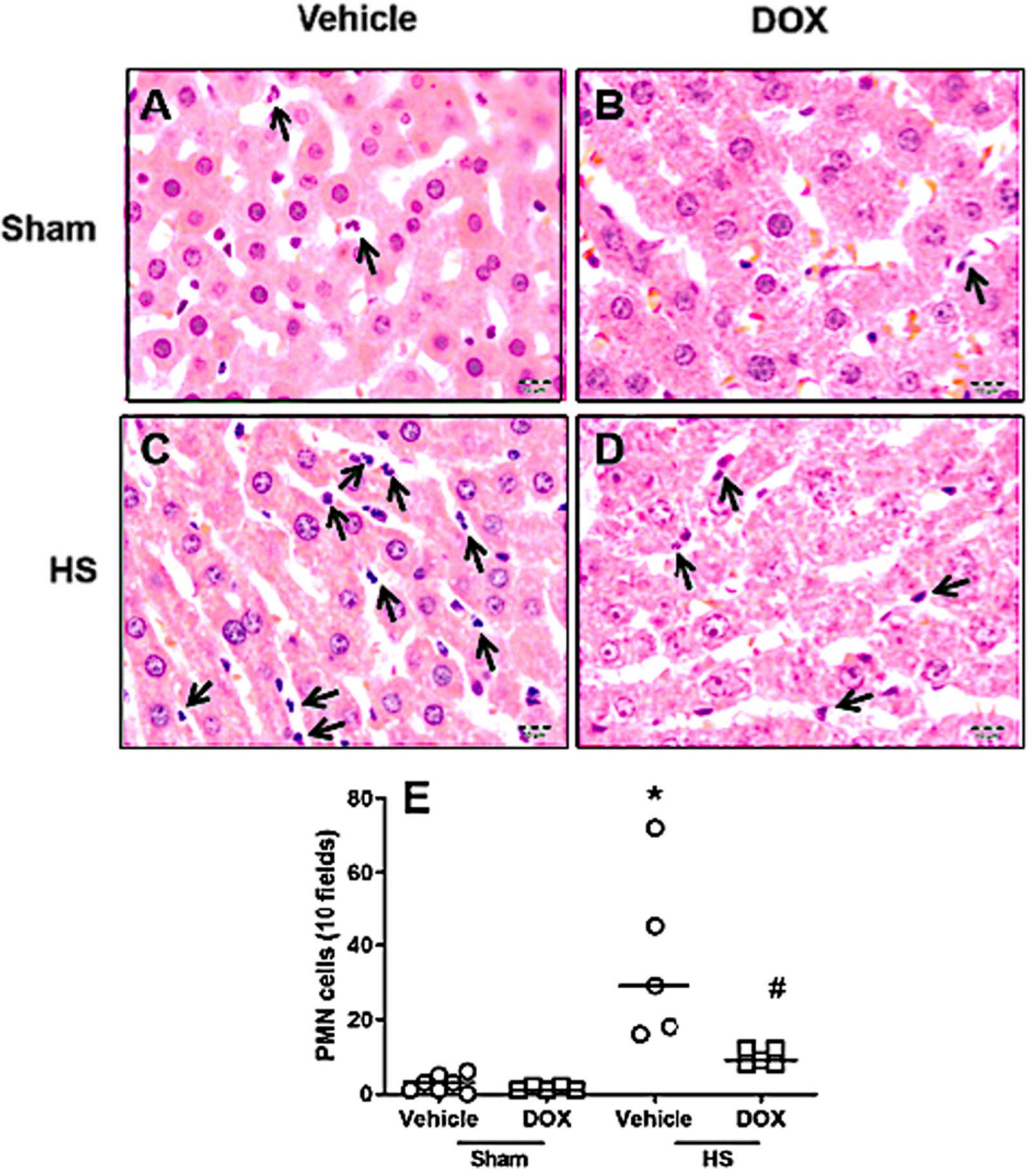
Representative image of liver stained with hematoxilin/eosin for control groups Sham (**A**, **B**) and HS treated with doxycycline (**B**, **D**) and results for number of PMN in all groups (**E**). Arrow indicate PMN in sinusoidal vessels. *P < 0.05 vs. sham + vehicle animals. ^#^P < 0.05 vs. HS + vehicle

Comparing the Sham group (Fig. 4A) to the HS group (Fig. 4C), the inflammatory cells into the liver were significantly increased in the HS group. However, after DOX treatment, the number of inflammatory cells was significantly reduced in the HS group (Fig. 4D, E).

## Discussion

The primary goal of this investigation was to assess the protective effect of DOX when given before resuscitation in a rat model of HS. In order to do that, blood markers were used to infer kidney, liver and cardiovascular dysfunctions as well as the degree of morphological damage caused by inflammation in the liver.

As expected, the HS condition caused a number of alterations in all parameters assessed, such as a decrease in renal blood flow and elevated plasma levels of urea and creatinine, which may indicate kidney dysfunction. Additionally, the elevated levels of AST, ALT, and CPK indicate that HS model was associated with liver and muscle injury.

HS pathogenesis is invariably associated with hypoxia and ischemia–reperfusion damage. Reactive oxygen species (ROS) generation rises as blood flows through an ischemic tissue, which triggers the release of more inflammatory mediators. Together, these mediators contribute to organ and cell damage, which results in multiple organ failure and dysfunction [21].

In our study, the model of HS employed was found to reproduce the organ dysfunction observed in patients that suffered severe hemorrhage. Importantly, DOX was administered as a treatment immediately preceding the resuscitation with blood. The timing of the treatment was aimed at enhancing the clinical relevance of our study. When we examined how DOX protects the advance of some signs of organ damage, we found that DOX treatment in HS rats reduced liver injury, renal dysfunction, and CPK, an indication of muscle damage. Also, the improvement of kidney dysfunction in HS rats was confirmed with the return of RBF to sham values. Our results corroborates and extends a previous study that evaluated the effect of DOX on an acute model of HS and showed that DOX reduces liver damage and kidney dysfunction [22]. However, the mouse terminal model of HS used by the authors had a 6-h outcome period, in contrast to our investigation, which focused on the effects of DOX 24 h after the injury (a recovery model). In this previous study, they also evaluated the death rate over a week and discovered that DOX provided persistent protection.

Neutrophils produce cytokines, ROS, and inflammatory response enzymes such as myeloperoxidase, all of which have been connected to organ damage and dysfunction. The protective effect of DOX on liver injury indicated by plasma markers such as AST and ALT may have been generated, at least in part, by the decrease in the number of inflammatory cells seen in the liver of HS rats treated with DOX. Previous studies have reported a protective role of DOX against ischemia and reperfusion injury in hepatocytes. They show that the cytoprotection was mediated by the inhibition of the mitochondrial calcium uniporter rather than MMP inhibition [23]. However, if in our model this was the mechanism for the attenuation of liver inflammatory process, it needs to be determined.

Treatment with DOX has been associated with an improvement of cardiovascular outcome due to its antioxidant effect (reviewed in [11]). As HS is associated with an increase of ROS, antioxidant therapies could be beneficial to prevent cardiovascular dysfunction. The fact that MAP and HR were similar across all groups suggests that DOX therapy had no negative impact on cardiovascular parameters evaluated in our study. However, contrary to our expectations, no improvement on the response to Ang II was observed in HS rats.

Interestingly, the metabolites of nitric oxide (NOx) were reduced in plasma of HS rats treated with DOX. In pathological conditions, NO is mainly derived from nitric oxide synthase (NOS-2), which has been previously shown to be an important mediator of organ injury in HS [7, 8] and also in other conditions such as sepsis [24–27]. The upregulation of NOS-2 expression is observed in the initial hours following both septic shock [27] and HS [28]. In both conditions, the nitric oxide (NO) derived from NOS-2 has been documented to play a role in cardiovascular dysfunction and uncontrolled inflammation. This suggests noteworthy parallels between the pathophysiological mechanisms of these two types of shock. Therefore, the NOx levels reduction by DOX indicates important protection for the injuries associated with HS and are in accordance with previous studies that showed the potential of DOX treatment to decrease NOx levels both by reducing the activity and expression of NOS-2 [12, 13].

Moreover, NOx levels were positively linked with the indicators of renal dysfunction, urea and creatinine, indicating some level of connection. However, no correlation was observed with the indicators of liver injury, AST and ALT, which suggests alternative protective mechanisms involving these markers that are not yet understood.

Some limitations of the study should be mentioned. First, the use of anaesthetic sodium thiopenthone. Anaesthetics used in animal experimentation interferes with several parameters. Barbiturates may have effects on physiological parameters and the recovery is marked by a prolonged period before full consciousness is restored. Therefore, the use of different anaesthetics imposes limitations when comparing studies [29]. Another limitation was the use of crystalloid during resuscitation. Although we used the shed blood for resuscitation, a small amount of crystalloid was also administrated. There is a long discussion about what is the best fluid for resuscitation. Balanced crystalloid offers theoretic benefits over normal saline; however, large studies demonstrate only minimal benefits [30]. A recent meta-analysis pointed that among sepsis and surgical patients, balanced crystalloid and albumin attained lower mortality rates and lower risk of acute kidney injury than saline and low molecular weight hydroxyethyl starch. However, balanced crystalloids required the greatest fluid resuscitation volume than all other fluid types, and there are clear harms associated with the aggressive use of fluid administration [31]. Therefore, the choice of the anaesthetics and the fluids may interfere with the outcome and our results should be interpreted with caution when comparing to other studies.

## Conclusion

In summary, our findings show that DOX protected liver and kidney against injury and dysfunction in a relevant HS model and could be a strategy to reduce organ damage associated with ischemia-and-reperfusion injury.

## Data Availability

The datasets used and/or analyzed during the current study are available from the corresponding author on reasonable request.
